# Cancer screening adherence among e-cigarette users in the United States

**DOI:** 10.18332/tpc/207098

**Published:** 2025-07-25

**Authors:** Areesh Mevawalla, Mujtaba Khalil, Zayed Rashid, Abdullah Altaf, Azza Sarfraz, Timothy M. Pawlik

**Affiliations:** 1 Department of Surgery, The Ohio State University Wexner Medical Center and James Comprehensive Cancer Center, Columbus, United States

**Keywords:** oncology, smoking status, cancer screening, BRFSS, e-cigarettes

## Abstract

**INTRODUCTION:**

Tobacco use patterns have dramatically shifted, with electronic cigarettes (e-cigarettes) rapidly increasing in popularity despite uncertainty about their health impacts. This study examines adherence to preventive cancer screening guidelines among cigarette smokers, e-cigarette users, and non-smokers, addressing a critical gap in understanding how tobacco use influences engagement in preventive healthcare.

**METHODS:**

A total of 445132 adult respondents were queried from the 2022 Behavioral Risk Factor Surveillance System data. Analytic samples were restricted to age- and sex-eligible sub-cohorts for each cancer type, therefore including adults 50–75 years, women 50–74 years, and women 21–65 years for colon, breast and cervical screening, respectively. Within these analytic samples, we assessed the association between socioeconomic characteristics, smoking status, and screening adherence using weighted logistic regression, adjusted for relevant factors.

**RESULTS:**

Screening adherence was lowest among cigarette smokers, intermediate among e-cigarette users, and highest among non-smokers. Compared to non-smokers, cigarette smokers had significantly lower odds of adhering to colorectal (AOR=0.75; 95% CI: 0.58–0.81), breast (AOR=0.57; 95% CI: 0.52–0.61), and cervical cancer screening (AOR=0.67; 95% CI: 0.62–0.72). E-cigarette users also showed reduced adherence to colorectal (AOR=0.90; 95% CI: 0.81–0.95) and breast cancer screening (AOR=0.75; 95% CI: 0.70–0.81) but not cervical cancer screening.

**CONCLUSIONS:**

E-cigarette users exhibited suboptimal adherence to recommended cancer screenings, suggesting that perceptions of reduced risk associated with vaping do not translate into improved preventive healthcare behaviors. Targeted public health initiatives addressing risk misconceptions and healthcare access barriers are necessary to improve screening rates among all tobacco users.

## INTRODUCTION

Tobacco use in the United States (US) is changing rapidly with new methods of consumption gaining popularity^1^. Conventional cigarette smoking has declined to a historic low with only about 12% of adults consuming tobacco in this manner; in contrast, e-cigarette use has surged with roughly 7% of adults and nearly one in five young adults (aged 18–24 years) using these devices^2^. E-cigarettes have been promoted as a safer alternative and as a possible smoking cessation tool, but these benefits have been hotly debated^3^. Although initially viewed as lower risk, emerging evidence has demonstrated that e-cigarettes are as harmful as, or even more harmful, than smoking^3^. While much of the public discourse has been focused on the health effects of e-cigarettes, the impact of shifting perceptions relative to tobacco consumption habits on other important preventive health behaviors, such as cancer screening, remains unclear.

Cancer screening for breast, cervical, colorectal, and lung cancers has been demonstrated to be effective in reducing mortality; however, cancer screening among traditional tobacco users has been suboptimal, with cigarette smokers being less likely to complete recommended cancer screenings^4^. Most evidence on screening comes from US based studies, specifically, smokers have 20–50% lower odds of undergoing colonoscopy, mammography, or Pap testing^5^. International data, although more limited, suggest similar trends in other high-income settings^6^. As a result, cancers, even non-lung tumors, are more likely to be diagnosed at later stages among smokers. Disparities in screening and cancer stage at diagnosis likely reflect a combination of behavioral and structural barriers^7^. Of note, smokers more frequently report fatalism, stigma, and mistrust in healthcare settings, which may contribute to screening avoidance^8^. Additionally, smoking is more prevalent among socioeconomically disadvantaged populations, which often experience limited access to insurance, transportation, and routine healthcare^9^. In turn, communities experiencing high social and environmental hardship often have lower screening completion with concomitant higher cancer mortality^10^.

With the increasing marketing and use of e-cigarettes as ‘lower risk tobacco alternatives’, it is important to understand how e-cigarette users perceive engagement with preventive cancer care. Some e-cigarette users may be health-motivated former smokers, while other individuals, especially younger adults who have never smoked, may underestimate their risks and avoid preventive care^11^. To date, whether e-cigarette use impacts the likelihood of patients to engage in cancer prevention behaviors remains unknown. Therefore, the objective of the current study was to define screening adherence among cigarette smokers, e-cigarette users, and non-smokers using nationally representative data. By analyzing adherence to United States Preventive Services Task Force (USPSTF)-recommended colorectal, breast, and cervical cancer screenings, we sought to define the impact of e-cigarette use on population-based cancer screening compliance.

## METHODS

### Data source, sample selection and outcome

This cross-sectional study utilized publicly available, de-identified data from the 2022 Behavioral Risk Factor Surveillance System (BRFSS), a nationally representative survey administered by the Centers for Disease Control and Prevention (CDC)^12^. BRFSS collects information on health-related behaviors, including questions on both e-cigarette and cigarette use among non-institutionalized adults aged ≥18 years. Among 445132 respondents, those reporting a prior history of any cancer were excluded, and three analytic sub-samples were defined according to USPSTF age and sex specific eligibility criteria: adults aged 45–75 years for colorectal cancer screening (n=181379), women aged 40–74 years for breast cancer screening (n=20157), and women aged 21–65 years without a history of hysterectomy for cervical cancer screening (n=19152). Smoking status was grouped into three categories. E-cigarette users were respondents who reported ever using an e-cigarette or other electronic vaping product and currently using it every day or some days^13^. Cigarette smokers were defined as respondents who reported currently smoking cigarettes every day or some days, regardless of e-cigarette use. Individuals who reported dual use (i.e. currently smoking cigarettes and using e-cigarettes) were included in the cigarette smoker category, as their behavioral profiles and cancer risk patterns align more closely with combustible tobacco use^14,15^. Non-smokers were respondents who had never used e-cigarettes and reported smoking cigarettes ‘not at all’ at the time of the survey; this category therefore included both lifetime never smokers and former cigarette smokers.

The primary outcome was screening adherence, defined as being up-to-date with USPSTF-recommended cancer screening intervals for colorectal, breast, or cervical cancer (Supplementary file Table 1)^16^. Respondents were classified as adherent if they reported having received the recommended screening test within the guideline-specified time frame. Those who did not meet screening criteria despite being eligible were classified as non-adherent. This binary adherence outcome was assessed separately for each cancer type. This study followed the Strengthening the Reporting of Observational Studies in Epidemiology (STROBE) reporting guideline for observational studies^17^. This study was determined to be exempt from ethical approval by The Ohio State University Institutional Review Board, due to the use of publicly available, de-identified data.

### Covariates and outcomes of interest

Covariates included age, sex, race and ethnicity, education level, rurality (metropolitan vs non-metropolitan residence), employment status, annual household income, and access to primary care. Smoking behavior, including both cigarette and e-cigarette use, was also assessed. Heavy alcohol use was defined as more than 14 drinks per week for men and more than 7 drinks per week for women^12^. Sex was self-reported and categorized based on sex assigned at birth (male or female)^18^. Race and ethnicity were grouped as non-Hispanic White, non-Hispanic Black, Hispanic, and Other (including Native Hawaiian or Pacific Islander, American Indian or Alaska Native, and non-Hispanic Asian). Education was categorized as less than high school, high school graduate, or college graduate^19^. Employment status was classified as employed or unemployed, whereas household income was categorized as low (<$50000) versus high (≥$50000)^20,21^.

The primary outcome was adherence to USPSTF-recommended cancer screening guidelines among e-cigarette users compared with cigarette smokers and non-smokers. Secondary outcomes included association between screening adherence and sociodemographic variables including, age, race and ethnicity, education level, income, marital status, residence (urban, rural), and having a primary care provider (PCP) visit within the last year.

### Statistical analysis

Descriptive statistics were summarized as counts with percentages for categorical variables. Group differences were assessed using the chi-squared or Fisher’s exact test for categorical variables. Descriptive results are presented for the overall analytic sample and stratified by smoking status (e-cigarette users, cigarette smokers, and non-smokers) and cancer screening adherence.

For each cancer type, multivariable logistic regression models were used to evaluate the association between smoking status (reference: non-smokers) and screening adherence (adherent= 1, non-adherent=0). Covariates were selected *a priori* to capture key sociodemographic and access-to-care factors and were included simultaneously. These included age categories, race/ethnicity, education level, annual household income, marital status, employment status, metropolitan versus non-metropolitan residence, and history of primary care provider (PCP) visit in the past year. Adjusted odds ratios (AORs) with 95% confidence intervals (CIs) are reported. To assess the robustness of associations within socioeconomically advantaged populations, sub-analysis included models restricted to: 1) respondents in the highest household income quartile; and 2) respondents with education level beyond college. All tests were two-sided and p<0.05 considered statistically significant. All analyses were conducted using Stata version 18.0 (StataCorp LLC).

## RESULTS

### Descriptive statistics

A total of 445132 adults were included in the initial sample (Table 1). Most respondents were non-Hispanic White (n=333514; 74.9%), aged ≥65 years (n=161451; 36.3%), metropolitan residents (n=318082; 73.0%), and college graduates (n=307748; 69.1%); more than half reported annual household income <$50000 (n=203945; 54.2%). The majority of respondents were publicly insured (46.3%), while 9.2% had no insurance. Approximately 9 out of 10 respondents were non-smokers, with 5.0% e-cigarette users and 4.3% cigarette smokers. Of note, e-cigarette users were younger (aged 25–34 years: 25.6% vs 13.8%), more often male (54.0% vs 51.4%) and college-educated (58.2 % vs 52.4%), resided in metropolitan areas (75.6% vs 68.3%) and were more likely to earn ≥$50000 annually (42.6% vs 33.9%) compared with cigarette smokers (all p<0.001). Furthermore, e-cigarette users were less likely to have visited a primary-care provider in the past year than cigarette smokers (64.6% vs 69.2%; p<0.001).

**Table 1:** Demographic and socioeconomic characteristics of US adults, overall and by smoking status, 2022 BRFSS (N=445132)

*Characteristics*	*Total* *n (%)*	*Cigarette smokers* *n (%)*	*E-cigarette smokers* *n (%)*	*Non-smokers* *n (%)*	*P^a^*
**Total**	445132 (100)	19425 (4.3)	22116 (5.0)	403591 (90.7)	
**Age** (years)					<0.001
18–24	26943 (6.1)	548 (5.3)	4999 (22.6)	21396 (5.3)	
25–34	47840 10.8)	2685 (13.8)	5650 (25.6)	39505 (9.8)	
35–44	59174 (13.3)	4097 (20.8)	4430 (20.0)	50697 (12.6)	
45–54	66984 (15.1)	4003 (20.6)	2951 (13.3)	60030 (14.9)	
55–64	82740 (18.6)	4517 (23.3)	2267 (10.3)	75956 (18.8)	
≥65	161451 (36.3)	3625 (18.7)	1819 (8.2)	156007 (38.7)	
**Sex**					<0.001
Male	37441 (47.1)	1929 (51.4)	2270 (54.0)	33242 (46.5)	
Female	41456 (52.2)	1811 (48.2)	1906 (45.3)	37739 (52.8)	
Unknown	366235	15685	17940	332610	
**Ethnicity**					<0.001
White	333514 (74.9)	14665 (75.5)	15889 (71.8)	302960 (75.1)	
Black	35876 (8.1)	1501 (7.7)	1432 (6.5)	32943 (8.2)	
Asian	13487 (3.0)	316 (1.6)	678 (3.1)	12493 (3.1)	
American Indian	7120 (1.6)	556 (2.9)	474 (2.1)	6090 (1.5)	
Hispanic	42977 (9.7)	1473 (7.6)	2437 (11.0)	39067 (9.7)	
Other	12158 (2.7)	914 (4.7)	1206 (5.5)	10038 (2.5)	
**Area**					<0.001
Metropolitan	318082 (73.0)	13068 (68.3)	16431 (75.6)	288583 (73.1)	
Non-metropolitan	117642 (23.0)	6080 (31.8)	5345 (24.6)	106217 (26.9)	
**Education level**					<0.001
≤High school	2383 (0.3)	67 (0.3)	61 (0.3)	2255 (0.6)	
High school graduate	135001 (30.3)	9173 (47.2)	9187 (41.5)	116641 (28.9)	
College graduate	307748 (69.1)	10185 (52.4)	12868 (58.2)	284695 (70.5)	
**Alcohol consumption**					
Yes	26305 (5.9)	2627 (13.5)	3084 (14.0)	20594 (5.1)	<0.001
**Annual household income** ($)					
<50000	241187 (54.2)	12849 (66.2)	12702 (57.4)	215636 (53.4)	<0.001
≥50000	203945 (45.8)	6576 (33.9)	9414 (42.6)	187955 (46.6)	
**Insurance type**					
Private	198319 (44.6)	7068 (36.4)	10580 (47.8)	180671 (44.8)	<0.001
Public	205910 (46.3)	9688 (50.0)	8230 (37.2)	187992 (46.6)	
Uninsured	40903 (9.2)	2669 (13.7)	3306 (14.9)	34928 (8.7)	
**PCP visit** (within 1 year)					
Yes	350944 (78.8)	13450 (69.2)	14296 (64.6)	323198 (80.1)	<0.001
**Self-reported general health**					
Good	424193 (95.3)	17584 (90.5)	20943 (94.7)	385666 (95.6)	<0.001

a Chi-squared.

Among breast, colon, and cervical screening, screening adherence was 71.5% (n=14419), 61.3% (n=111151), and 48.0% (n=9198), respectively. Overall, non-smokers were more likely to have adhered to recommended cancer screening across all cancer types (breast: 76.2%; colon: 63.9%; cervical: 69.7%). Notably, e-cigarette users were more likely to have adhered to screening for breast (12.5% vs 11.3%), colon (57.6% vs 50.8%), and cervical cancer screening (17.7% vs 12.6%) compared with cigarette smokers. Screening adherence was more likely with increasing age for breast (55–64 years: 31.2% vs 27.2%; ≥65 years: 36.7% vs 25.0%), and colon cancer (55–64 years: 67.5% vs 32.5%; ≥65 years: 71.1% vs 28.9%), while screening non-adherence was more prevalent among respondents aged 55–64 years for cervical cancer (29.8% vs 34.2%) (all p<0.001). Additionally, higher screening adherence across all cancers was observed among respondents reporting an annual household income ³$50000 and those with college education level (all p<0.001) (Supplementary file Tables 2–4).

### Sociodemographic factors associated with cancer screening

Several economic and social factors were associated with cancer screening adherence on multivariable analysis. Employment status was associated with lower odds of screening for colon cancer (AOR=0.94; 95% CI: 0.91–0.97), yet higher odds for breast (AOR=1.13; 95% CI: 1.04–1.24) and cervical (AOR=1.18; 95% CI: 1.09–1.28) cancer screening. Respondents who reported being married were associated with 29% and 34% greater odds of undergoing screening for colon (AOR=1.29; 95% CI: 1.10–1.19) and breast (AOR=1.34; 95% CI: 1.23–1.46) cancer, respectively. A history of recent PCP visit was strongly associated with higher odds of screening across all cancer types (colon: AOR=2.72; 95% CI: 2.63–2.82; breast: AOR=3.96; 95% CI: 3.61–4.34; cervical: AOR=1.80; 95% CI: 1.65–1.97). Additionally, household income <$50000 was associated with 28% lower odds of colon screening (AOR=0.72; 95% CI: 0.70–0.75), 27% decreased odds of breast screening (AOR=0.73; 95% CI: 0.66–0.80), as well as 31% reduced likelihood of cervical screening adherence (AOR=0.69; 95% CI: 0.63–0.75) (Table 2). Importantly, college education level demonstrated an association with overall increased screening adherence (colon: AOR=2.00; 95% CI: 1.87–2.12; breast: AOR=1.48; 95% CI: 1.23–1.77; cervical, AOR=1.81; 95% CI: 1.50–2.20).

**Table 2:** Multivariable logistic regression of sociodemographic and access-to-care factors associated with cancer screening adherence, by cancer type

*Variables*	*Colon cancer* *(N=181379)*	*Cervical cancer* *(N=19152)*	*Breast cancer* *(N=20157)*
		*AOR (95% CI)*	
**Employment status**			
Not employed ®	1	1	1
Employed	0.94 (0.91–0.97)*	1.18 (1.09–1.28)*	1.13 (1.04–1.24)*
**Marital status**			
Unmarried ®	1	1	1
Married	1.29 (1.24–1.34)*	1.03 (0.94–1.12)*	1.34 (1.23–1.46)*
**Residence**			
Urban ®	1	1	1
Rural	0.91 (0.89–0.94)*	0.99 (0.92–1.07)	0.92 (0.85–0.99)*
**PCP visit** (within 1 year)			
No ®	1	1	1
Yes	2.72 (2.63–2.82)*	1.80 (1.65–1.97)*	3.96 (3.61–4.34)*
**Annual household income** ($)			
≥50000 ®	1	1	1
<50000	0.72 (0.70–0.75)*	0.69 (0.63–0.75)*	0.73 (0.66–0.80)*
**Education level**			
≤High school ®	1	1	1
High school graduate	1.55 (1.46–1.65)*	1.02 (0.84–1.24)*	1.17 (0.97–1.40)*
College graduate	2.0 (1.87–2.12)*	1.81 (1.50–2.20)*	1.48 (1.23–1.77)*

AOR: adjusted odds ratio. PCP: primary care physician. ® Reference categories.

* p<0.05.

### Smoking status and cancer screening

On adjusted multivariable analysis, smoking status was independently associated with screening adherence across all cancer types. Specifically, e-cigarette users had 10% reduced odds for colorectal (AOR=0.90; 95% CI: 0.81–0.95) and 25% for breast cancer screening (AOR=0.75; 95% CI: 0.70–0.81), while odds of cervical cancer screening was comparable to non-smokers (p=0.30). Of note, cigarette smokers had the lowest odds of screening adherence for colorectal (AOR=0.75; 95% CI: 0.58–0.81), breast (AOR=0.57; 95% CI: 0.52–0.61), and cervical (AOR=0.67; 95% CI: 0.62–0.72) cancer (Table 3).

**Table 3:** Multivariable logistic regression of smoking status and cancer screening adherence

*Smoking status*	*Colorectal cancer* *(N=181379)* *AOR (95% CI)*	*Breast cancer* *(N=20157)* *AOR (95% CI)*	*Cervical cancer* *(N=19152)* *AOR (95% CI)*
**Never smokers** ®	1	1	1
**Cigarette smokers**	0.79 (0.72–0.78)*	0.57 (0.52–0.61)*	0.67 (0.62–0.72)*
**E-cigarette users**	0.90 (0.85–0.93)*	0.75 (0.70–0.81)*	0.99 (0.95–1.03)

AOR: adjusted odds ratio. All models are adjusted for age, ethnicity, education level, annual household income category, marital status, employment status, rural vs metropolitan residence, and primary care provider visit within the past year. The colorectal model includes adults aged 50–75 years; the breast model includes women aged 50–74 years; and the cervical model includes women aged 21–65 years without a history of hysterectomy. ® Reference category.

* p<0.05.

### Sensitivity analyses within socioeconomically advantaged subgroups

**Figure 1:**
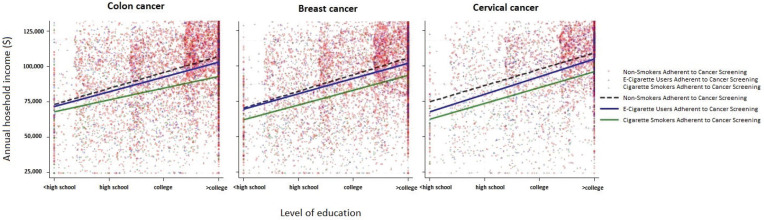
Scatter plot demonstrating the association between education level, household income, and cancer screening adherence, with trend lines corresponding to non-smokers, e-cigarette users, and cigarette smokers

Among individuals who completed age- and sex-appropriate cancer screening, adherence rates increase with both education and income. However, for the same education and income level, non-smokers were consistently more likely to have adhered to guideline recommended screening, followed by e-cigarette users and finally cigarette smokers, thus demonstrating that disparities in screening based on smoking status were evident across all socioeconomic groups (Figure 1).

Sensitivity analyses among respondents reporting high household income (Supplementary file Table 5) and college education or higher (Supplementary file Table 6) demonstrated that cigarette smoking remained significantly associated with lower colorectal screening adherence (high-income: AOR=0.74; 95% CI: 0.70–0.78; college-educated: AOR=0.72: 95% CI: 0.67–0.78) and breast cancer screening adherence (high-income: AOR=0.54; 95% CI: 0.46–0.64; college-educated: AOR=0.51; 95% CI: 0.42–0.63). In addition, e-cigarette users had significantly lower adherence to colorectal (high-income: AOR=0.88; 95% CI: 0.84–0.92; college-educated: AOR=0.89; 95% CI: 0.84–0.94) and breast cancer screening (high-income: AOR=0.71; 95% CI: 0.62–0.83; college-educated: AOR=0.77; 95% CI: 0.64–0.92).

### Sensitivity analysis: participants with recent primary care visit

To assess whether access to healthcare influenced our findings, we conducted sensitivity analyses restricted to individuals reporting at least one primary care provider (PCP) visit within the past year (Supplementary file Table 7). Adjusted multivariable logistic regression demonstrated persistently lower screening adherence among cigarette smokers compared to never smokers for colorectal (AOR=0.79; 95% CI: 0.76–0.82), breast (AOR=0.58; 95% CI: 0.52–0.66), and cervical cancers (AOR=0.86; 95% CI: 0.76–0.97; all p<0.001). Similarly, e-cigarette users exhibited significantly reduced odds for colorectal (AOR=0.90; 95% CI: 0.86–0.93) and breast cancer screening (AOR=0.83; 95% CI: 0.73–0.94), while no significant difference was observed for cervical cancer screening adherence (AOR=1.03; 95% CI: 0.92–1.15). These findings underscore that disparities in cancer screening adherence related to smoking status remained robust and were not explained by differential access to primary care services.

## DISCUSSION

Cancer screening has been linked with reduced cancer-related morbidity and mortality through early detection and timely treatment. Despite well-established guidelines, adherence with cancer screening remains suboptimal in certain populations^22^. The growing popularity of e-cigarettes, often perceived as less harmful than conventional cigarettes, may impact behaviors around preventive health^13^. Whether cancer screening adherence is different among e-cigarette users compared with cigarette smokers and non-smokers had not been previously investigated. The current study was therefore important as we leveraged large nationally representative data from the BRFSS database to evaluate cancer screening behaviors among e-cigarette smokers and non-smokers. Cigarette smokers demonstrated the lowest adherence rates for colorectal, breast, and cervical cancer screenings, while non-smokers had the highest adherence. Of note, e-cigarette users were younger and predominantly male. In addition, e-cigarette users had an intermediate screening adherence profile that more closely aligned with smokers than non-smokers. Collectively, these findings challenge the perception that transitioning to e-cigarettes inherently encourages healthier behaviors, highlighting that e-cigarette users remain at high risk of non-compliance with recommended cancer screenings.

The lower cancer screening participation among smokers and e-cigarette users may be understood, in part, through the lens of health beliefs and risk perceptions. Smokers, who often experience the highest cancer risk, paradoxically utilize screening less than never smokers. Quaife et al.^23^ noted that current smokers had lower odds of ever receiving a colonoscopy or mammogram versus never smokers, and were also less likely to be up-to-date with Pap testing. Behavioral research suggests that smokers often harbor fatalistic attitudes about cancer (e.g. believing that a serious diagnosis is inevitable or not preventable) and may fear finding out they are ill, which can deter proactive screening participation^24^. Additionally, many smokers downplay the benefits of early detection or feel stigmatized in healthcare settings, further reducing screening engagement. Interestingly, e-cigarette users frequently believe that they have mitigated their health risks by avoiding conventional smoking. In fact, survey data indicate that approximately two-thirds of adult e-cigarette users consider e-cigarettes to be less harmful than combustible cigarettes^25^. Early e-cigarette advertising and social-media campaigns framed e-cigarettes as a ‘safer’ or ‘cleaner’ alternative to combustible smoking by emphasizing reduced tar and odor rather than potential long-term health risks; this approach might have created a false sense of security regarding e-cigarette associated cancer risk^26^. In fact, recent experimental work showed that young adults exposed to such harm-reduction messages reported decreased perception of the cancer risk and diminished intention to obtain age-appropriate screening^27^. Our finding that exclusive e-cigarette users are less adherent than non-smokers, even after adjusting for socioeconomic factors, suggests that these risk-minimizing narratives may foster complacency toward preventive care. Public health campaigns should therefore pair e-cigarette cessation messaging with explicit reminders that e-cigarette use does not eliminate the need for guideline-concordant cancer screening. This is particularly important because many smokers adopt e-cigarettes as a cessation aid, a well-meaning substitution that may inadvertently lull users into neglecting other preventive behaviors^28^. Together, cancer fatalism among smokers and risk underestimation among e-cigarette users may help explain why both groups lag in screening uptake despite elevated cancer risk.

Beyond individual health beliefs, structural inequities, healthcare access barriers, and a diminished preventive orientation likely underline the lower cancer screening rates noted among smokers and e-cigarette users^29^. In the current study, both groups of individuals demonstrated characteristics associated with reduced engagement in preventive healthcare, with e-cigarette users more often being uninsured, which was consistent with prior data noting that e-cigarette users were twice as likely to lack health coverage compared with non-users^30^. This insurance gap may be a critical factor, as health coverage and a regular source of primary care are among the strongest predictors of receiving age-appropriate cancer screening. For example, older men who used e-cigarettes have been reported to be less likely to undergo PSA screening than both never smokers and traditional smokers^31^. Socioeconomic disadvantage compounds these challenges, as both smoking and, to a less extent, e-cigarettes are more prevalent among patients with lower income and lower education – populations that have traditionally experienced barriers to preventive care. (Figure 1). To this point, in the current study, e-cigarette users had lower median income and education level than non-smokers. These disparities translate into obstacles – financial strain, food insecurity, limited transportation, and lack of social support – all of which have been linked to missed screenings for colorectal, breast, and cervical cancers^32^. Gender norms may further influence screening behaviors, as e-cigarette use was more common in men who are generally less likely than women to engage in preventive services. Consistent with this finding, women in the current study were more likely to be adherent with colon cancer screening than men, mirroring national trends^33^. These patterns are magnified among uninsured and rural residents, where scarcity of clinics and travel costs intersect with the lower primary-care engagement we observed among e-cigarette users. Targeted strategies, mobile screening units, mailed FIT kits, and community health-worker navigation have been shown to reduce gaps in screening prevalence and the incorporation of systematic tobacco-use assessment may ensure that both combustible and e-cigarette users in underserved areas receive equitable access to cancer screening^34^.

The current study provides new insights into preventive health behaviors among e-cigarette users; an emerging population at high risk of underutilizing life-saving cancer screenings. Clinically, both current and former smokers, including those who have switched to e-cigarette use, should be prioritized for intensified outreach: embedding cancer-screening reminders into smoking-cessation or e-cigarette use education programs is a pragmatic approach. Because many adopt e-cigarettes to reduce harm, there is an opportunity to counter any false sense of security by emphasizing that ‘less harmful’ does not mean ‘harmless’ and that adherence to age-appropriate screenings remains essential. Public-health campaigns could similarly integrate messaging to remind all adults, especially smokers and e-cigarette users, of the benefits of recommended screenings. Structural barriers also demand attention: overlapping social vulnerabilities (e.g. low insurance coverage, rural residence, financial strain) among smokers and e-cigarette users mean that simple recommendations may not suffice. Our findings further underscore the importance of ongoing surveillance as the e-cigarette use population ages. BRFSS data show that current e-cigarette use is highest among adults aged 25–34 years, with prevalence declining after 44 years. As this population enters USPSTF screening windows, it will be vital to monitor whether their screening rates improve or remain low. Without targeted risk communication, such as incorporating e-cigarette use history into electronic health records and clinician reminders, e-cigarette users may continue to lag in adherence. Given uncertainties around long-term health effects of e-cigarette use, ensuring that these individuals stay up to date with preventive services will be increasingly important as they transition into older age groups.

### Limitations

Despite several strengths, including a large, nationally representative sample, these findings should be interpreted in light of several limitations. First, the cross-sectional design captures associations at a single point in time and therefore precludes causal inference. Second, all key variables – including cancer-screening completion and smoking/e-cigarette status – were self-reported, introducing potential recall error and social-desirability bias (e.g. over-reporting adherence or under-reporting tobacco use). Third, the 2022 BRFSS excluded adults without telephone access or with limited English/Spanish proficiency, and our analyses were further restricted to respondents who completed both the tobacco-use and cancer-screening modules, which may introduce selection bias. Fourth, only 17.7% of respondents reported their sex, and females comprised <10% of the analytic sample; because of this high level of missingness, sex was not included as a covariate in the multivariable models. Consequently, the results may not be fully generalizable to the broader US population, and sex-specific conclusions should be interpreted with caution. Moreover, since BRFSS is limited to the US, our findings may not be generalizable to populations in other countries or regions. BRFSS does not capture psychosocial determinants (e.g. health literacy, fatalism) that may confound both smoking and screening behaviors, and the cross-sectional design precludes establishing whether changes in tobacco use occurred before or after screening, limiting causal inference. Finally, although we adjusted for multiple sociodemographic and access-to-care factors, residual confounding from unmeasured variables (e.g. health attitudes or healthcare-seeking behavior) remains possible.

## CONCLUSIONS

Both cigarette smokers and e-cigarette users in the US had lower compliance with recommended cancer screenings. E-cigarette users, despite avoiding tobacco smoke, did not demonstrate an improved screening profile compared with smokers. These findings highlight the need for targeted interventions that integrate tobacco cessation efforts with preventive health promotion. By understanding the behavioral, perceptual, and systemic barriers facing smokers and e-cigarette users, clinicians and public health practitioners can better design programs to improve cancer screening uptake in these at-risk groups – ultimately aiming to reduce preventable cancer morbidity and mortality without overstating risks or benefits.

## Supplementary Material



## Data Availability

The data for this study were obtained from Centers for Disease Control and Prevention’s Behavioral Risk Factor Surveillance System. This is a publicly available database and can be accessed at https://www.cdc.gov/brfss/annual_data/annual_2022.html
