# Supplementary material for: Cancer screening adherence among e-cigarette users in the United States
Source: Tob Prev Cessat. 2025 Jul 25;11:10.18332/tpc/207098. doi: 10.18332/tpc/207098 (PMC12290923; doi:10.18332/tpc/207098)
Supplement: Supplementary file 1 [file TPC-11-34-s1.pdf]

Supplementary Table 1: Screening Eligibility Criteria

Cancer Type	Eligible Population	Recommended Screening Modalities
Colorectal Cancer	Adults aged 45–75 years	1. Fecal occult blood test (FOBT) annually<br>2. Fecal immunochemical test (FIT) annually<br>3. FIT-DNA every 1–3 years<br>4. CT colonography or sigmoidoscopy every 5 years<br>5. Colonoscopy every 10 years
Breast Cancer	Women aged 40–74 years	1. Screening mammography every 2 years 50-74<br>2. Optional to start at 40 till age 49 according to risk discussion.
Cervical Cancer	Women aged 21–65 years with a cervix	1. Ages 21–29: Cytology (Pap test) every 3 years<br>2. Ages 30–65: One of the following: Cytology every 3 years– hrHPV testing every 5 years– Co-testing every 5 years

Supplementary Table 2: Demographic Characteristics of Women Eligible for Breast Cancer Screening by Screening Adherence Status (n = 20,157; BRFSS 2022 respondents aged 40–74 years)

	**Screening Adherence**[1]	**Screening non-adherence**[1]	**p value**[2]
**Breast cancer screening**	14,419(71.5%)	5,738 (28.5%)	<0.001
**Age category(y)**			
35–44	1,252 (8.7%)	1,237 (21.6%)	< 0.001
45–54	3,373 (23.4%)	1,506 (26.3%)	
55–64	4,497 (31.2%)	1,562 (27.2%)	
≥65	5,297 (36.7%)	1,433 (25.0%)	
**Ethnicity**			
White	11,328 (78.6%)	4,374 (76.2%)	< 0.001
Black	689 (4.8%)	190 (3.3%)	
Asian	629 (4.4%)	188 (3.3%)	
American Indian	232 (1.6%)	144 (2.5%)	
Hispanic	995 (6.9%)	582 (10.1%)	
Other	546 (3.8%)	260 (4.5%)	
**Smoking status**			
Non-smoker	10,991 (76.2%)	3,667 (63.9%)	< 0.001
E-cigarette user	1,799 (12.5%)	845 (14.7%)	
Cigarette smoker	1,629 (11.3%)	1,226 (21.4%)	
**Education level**			
< high school	3,501 (24.3%)	1,694 (29.6%)	< 0.001
High school graduate	4,106 (28.5%)	1,782 (31.1%)	
College graduate	6,804 (47.2%)	2,256 (39.4%)	
**Annual household income**			
<$50,000	3,713 (25.8%)	2,042 (35.6%)	< 0.001
≥$50,000	10,706 (74.2%)	3,696 (64.4%)	

[1]n(%)
[2]chi-square

Supplementary Table 3: Demographic Characteristics of Adults Eligible for Colorectal Cancer Screening by Screening Adherence Status (n = 181,379; BRFSS 2022 respondents aged 45–75 years)

	**Screening Adherence**[1]	**Screening non-adherence**[1]	**p value**[2]
**Colon Cancer Screening**	111,151 (61.3%)	70,228 (38.7%)	<0.001
**Age cateogories(y)**			
45–54	19,917 (39.9%)	30,005 (60.1%)	
55–64	42,713 (67.5%)	20,547 (32.5%)	
65+	48,521 (71.1%)	19,676 (28.9%)	<0.001
**Sex**			
Male	9,997 (60.2%)	6,623 (39.8%)	
Female	10,993 (61.7%)	6,837 (38.3%)	0.029
**Ethnicity**			
White	89,970 (63.1%)	52,545 (36.9%)	
Black	9,132 (63.1%)	5,350 (36.9%)	
Hispanic	1,824 (49.2%)	1,883 (50.8%)	
Asian	1,533 (50.5%)	1,502 (49.5%)	
American Indian	6,344 (47.9%)	6,903 (52.1%)	
Other	2,348 (53.4%)	2,045 (46.6%)	<0.001
**Smoking status**			
Non-smoker	84,762 (63.9%)	47,803 (36.1%)	
E-cigarette user	13,506 (57.6%)	9,928 (42.4%)	
Cigarette smoker	12,883 (50.8%)	12,497 (49.2%)	<0.001
**Level of education**			
< High school	27,459 (55.1%)	22,420 (44.9%)	
High school graduate	31,388 (61.7%)	19,457 (38.3%)	<0.001
College graduate	52,209 (64.9%)	28,225 (35.1%)	
**Annual household income**			
< $50,000	26,609 (54.5%)	22,172 (45.5%)	<0.001
≥ $50,000	84,542 (63.8%)	48,056 (36.2%)	

[1]n(%)
[2]chi-square

Supplementary Table 4: Demographic Characteristics of Women Eligible for Cervical Cancer Screening by Screening Adherence Status (n = 19,152; BRFSS 2022 respondents aged 21–65 years, no hysterectomy)

	**Screening Adherence**[1]	**Screening non-adherence**[1]	**p value**[2]
**Cervical cancer screening**	9,198 (48.0)	9,954 (52.0)	<0.001
**Age category(y)**			
18–34 years	1,494 (16.2%)	1,861 (18.7%)	
35–44 years	2,418 (26.3%)	2,329 (23.4%)	
45–54 years	2,544 (27.7%)	2,363 (23.7%)	
55–64 years	2,742 (29.8%)	3,401 (34.2%)	<0.001
**Ethnicity**			
White	6,891 (74.9%)	6,915 (69.5%)	
Black	429 (4.7%)	526 (5.3%)	
Asian	346 (3.8%)	541 (5.4%)	
American Indian	156 (1.7%)	224 (2.3%)	
Hispanic	938 (10.2%)	1,217 (12.2%)	
Other	438 (4.8%)	531 (5.3%)	<0.001
**Smoking Status**			
Non-Smoker	6,412 (69.7%)	6,444 (64.7%)	
E-Cigarette	1,625 (17.7%)	1,744 (17.5%)	<0.001
Cigarette	1,161 (12.6%)	1,766 (17.7%)	
**Education Level**			
< High School	1,718 (18.7%)	3,045 (30.6%)	
High School graduate	2,519 (27.4%)	2,905 (29.2%)	
College Graduate	4,955 (53.9%)	3,992 (40.2%)	<0.001
**Annual household income**			
< $50,000	1,869 (20.3%)	3,204 (32.2%)	
≥ $50,000	7,329 (79.7%)	6,750 (67.8%)	<0.001

[1]n(%)
[2]chi-square

Supplementary Table 5: Multivariable Logistic Regression of Factors Associated with Cancer Screening Adherence Among Adults with High Annual Household Income (>$50,000)

	Colon cancer (n=110,465)	Cervical cancer (n=11,933)	Breast cancer (n=11,827)
	Adjusted Odds ratio (95% CI)		
**Employment status**			
Not employed	Ref.	Ref.	Ref.
Employed	0.97 (0.94-1.01)	1.32(1.19-1.46)*	1.15(1.02-1.30)*
**Marital status**			
Un-married	Ref.	Ref.	Ref.
Married	1.14(1.10-1.19)*	1.09 (0.97-1.23)	1.20 (1.06-1.37)*
**Urbanicity**			
Urban	Ref.	Ref.	Ref.
Rural	0.93 (0.90-0.96)*	0.99 (0.90-1.10)	0.95 (0.86-1.05)
**PCP visit (within 1 yr)**			
No	Ref.	Ref.	Ref.
Yes	2.50 (2.37-2.56)*	1.85 (1.66-2.05)*	3.86 (3.43-4.35)*
**Smoking status**			
Non-smoker	Ref.	Ref.	Ref.
E-cigarette smoker	0.88(0.84-0.92)*	1.01 (0.89-1.14)	0.71 (0.62-0.83)*
Cigarette smoker	0.74(0.70-0.78)*	0.88 (0.75-1.02)	0.54 (0.46-0.64)*
**Education level**			
<high school	Ref.	Ref.	Ref.
High school graduate	1.95 (1.70-2.25)*	1.72 (1.07-2.78)*	2.49 (1.53-4.10)*
College graduate	2.15(1.71-2.46)*	2.24 (1.39-3.59)*	3.20 (1.97-5.20)*

Abbreviations: AOR, adjusted odds ratio; CI, confidence interval; Ref., reference category; PCP, primary care physician

*Indicate $p < 0.05$.

Notes:

Multivariable logistic regression models were used to estimate adjusted odds ratios (aORs) and 95% confidence intervals (CIs) for the likelihood of cancer screening adherence among adults reporting an annual household income greater than $50,000. Analyses were restricted to age- and sex-eligible respondents for each screening type: colorectal (ages 50–75), breast (women aged 50–74), and cervical (women aged 21–65 without a history of hysterectomy).

Cancer screening adherence was defined according to United States Preventive Services Task Force (USPSTF) guidelines in effect during each survey year.

All models were adjusted for age, race/ethnicity, education level, marital status, employment status, urban vs. rural residence, primary care provider (PCP) visit within the past 12 months, and smoking status (non-smoker, e-cigarette user, cigarette smoker).

Estimates account for the complex sampling design of the Behavioral Risk Factor Surveillance System (BRFSS) using survey weights, strata, and primary sampling units.

Supplementary Table 6: Multivariable Logistic Regression of Factors Associated with Cancer Screening Adherence Among College-Educated Adults

	Colon cancer (n= 80,434)	Cervical cancer (n= 8,947)	Breast cancer (n=9,060)
	Adjusted Odds ratio (95% CI)		
**Employment status**			
Not employed	Ref.	Ref.	Ref.
Employed	0.92 (0.89-0.97)*	1.24 (1.10-1.41)*	1.01 (0.87-1.17)
**Marital status**			
Un-married	Ref.	Ref.	Ref.
Married	1.09 (1.04-1.13)*	0.97 (0.85-1.11)	1.14 (0.99-1.31)
**Urbanicity**			
Urban	Ref.	Ref.	Ref.
Rural	0.93 (0.89-0.97)*	1.00 (0.90-1.11)	0.88 (0.78-0.99)*
**PCP visit (within 1 yr)**			
No	Ref.	Ref.	Ref.
Yes	2.40 (2.30-2.52)*	1.70 (1.51-1.93)*	3.72 (3.24-4.27)*
**Smoking status**			
Non-smoker	Ref.	Ref.	Ref.
E-cigarette smoker	0.89 (0.84-0.94)*	1.12 (0.97-1.31)	0.77 (0.64-0.92)*
Cigarette smoker	0.72 (0.67-0.78)*	0.83 (0.67-1.02)	0.51 (0.42-0.63)*
**Annual household income($)**			
<50,000	Ref.	Ref.	Ref.
>50,000	1.67 (1.53-1.82)*	1.60 (1.21-2.11)*	1.80 (1.40-2.33)*

Abbreviations: AOR, adjusted odds ratio; CI, confidence interval; Ref., reference category; PCP, primary care physician

*indicate $p < 0.05$.

Notes:

Multivariable logistic regression models were used to estimate adjusted odds ratios (aORs) and 95% confidence intervals (CIs) for the likelihood of cancer screening adherence among adults with a college degree. Analyses were restricted to age- and sex-eligible respondents for each screening type: colorectal (ages 50–75), breast (women aged 50–74), and cervical (women aged 21–65 without a history of hysterectomy). Cancer screening adherence was defined according to United States Preventive Services Task Force (USPSTF) guidelines in effect during each survey year.

All models were adjusted for age, race/ethnicity, annual household income, marital status, employment status, urban vs. rural residence, primary care provider (PCP) visit within the past 12 months, and smoking status (non-smoker, e-cigarette user, cigarette smoker).

Estimates account for the complex sampling design of the Behavioral Risk Factor Surveillance System (BRFSS) using survey weights, strata, and primary sampling units.

Supplementary table 7. Adjusted Odds of Cancer Screening Adherence by Smoking Status Among Adults with a Primary Care Visit in the Past Year

**Smoking Status**	**Colorectal cancer** (n=181,379) AOR (95% CI)	**Breast cancer** (n=20,157) AOR (95% CI)	**Cervical cancer** (n=19,152) AOR (95% CI)
**Never smokers**	Ref.	Ref.	Ref.
**Cigarette smokers**	0.79 (0.76–0.82)*	0.58 (0.52–0.66)*	0.86 (0.76–0.97)*
**E-cigarette users**	0.90 (0.86–0.93)*	0.83 (0.73–0.94)*	1.03 (0.92–1.15)

Abbreviations: AOR, adjusted odds ratio; CI, confidence interval; Ref., reference category. *indicate $p < 0.001$.

Notes:

1. All models are adjusted for age, ethnicity, level of education, annual household income category, marital status, employment status, rural vs. metropolitan residence, and primary care provider visit within the past year.
2. The colorectal model includes adults aged 50–75 years; the breast model includes women aged 50–74 years; and the cervical model includes women aged 21–65 years without a history of hysterectomy.

© 2025 Mevawalla A. et al.
